# A Comprehensive Overview of circRNAs: Emerging Biomarkers and Potential Therapeutics in Gynecological Cancers

**DOI:** 10.3389/fcell.2021.709512

**Published:** 2021-07-21

**Authors:** Yalan Ma, Lianwen Zheng, Yiyin Gao, Wenying Zhang, Qiang Zhang, Ying Xu

**Affiliations:** Department of Obstetrics and Gynecology, Reproductive Medical Center, The Second Hospital of Jilin University, Changchun, China

**Keywords:** circular RNA, expression, biomarkers, therapeutics, gynecological cancers

## Abstract

Circular RNA (circRNA) is a highly conserved, stable and abundant non-coding RNA (ncRNA). Also, some circRNAs play an essential part in the progression of human cancers. CircRNA is different from traditional linear RNA. CircRNA has a closed circular structure, so it is resistant to exonuclease-mediated degradation and is more stable than linear RNA. Numerous studies have found that many circRNAs can act as a microRNA (miRNA) sponge, interact with RNA-binding proteins, regulate gene transcription, affect alternative splicing and be translated into proteins. Recently, some studies have also indicated that circRNA participates in the progression of gynecological cancers. In addition, circRNA can act as a promising biomarker for the diagnosis of gynecological tumors. Additionally, they can also play a key role in the prognosis of gynecological tumors. Furthermore, to our delight, circRNA may be a potential therapeutic target in gynecological cancers and widely used in clinical practice. This article reviews the functions and related molecular mechanisms of circRNAs in gynecological tumors, and discusses their potential as biomarkers for diagnostic and prognostic and therapeutic targets for gynecological cancers.

## Introduction

Circular RNA (circRNA) is a unique class of RNA that unlike other RNAs forms a covalently closed loop, generally thought to be non-coding, which as a result of improved sequencing strategies has recently gained renew interest among scientists (Hsu and Coca-Prados, 1979; Ashwal-Fluss et al., 2014). Many scientists believe that circRNA is a widely distributed and diverse small endogenous RNA with multiple regulatory functions. According to the synthesis mechanism based on the location of the genome splice junction from which circRNAs originate, circRNAs are classified into four major types: exonic circRNAs (ecircRNAs), intronic circRNAs, exon-intron circRNAs (EIciRNAs), and intergenic circRNAs (Dong Y. et al., 2017). Unlike linear RNA, circRNA is a covalently closed single-stranded circular transcript without 5′cap or 3′poly(A) tail. This special structure makes the circRNA a stable, conserved, highly abundant RNA that is dynamically expressed in specific tissues through a unique process (Nicolet et al., 2018). Since circRNA has the advantages of cell- type-, tissue- and developmental stage-specific expression, and cancer cells have diverse expression profiles of the different types of circRNA, circRNA can also be used to classify and identify different tumor types (Athanasiadis et al., 2004; Salzman et al., 2013; Zhang et al., 2017; Nicolet et al., 2018; Smid et al., 2019). In recent years, many studies have indicated that circRNA is related to a variety of human diseases, including cardiovascular diseases (Hansen et al., 2013; Dong et al., 2017; Zhang et al., 2017; Smid et al., 2019), neurological diseases (Liu et al., 2017; Kristensen et al., 2019), and other diseases (Li P. et al., 2015; Wang et al., 2015). In addition, recently, it has been found that the development of certain tumors is affected by functional circRNA-mediated regulatory networks in various ways, such as acting as a microRNA (miRNA) “sponge” and regulating the function of miRNA target genes (Hansen et al., 2013). Besides, it has been reported that certain circRNAs can bind with specific RNA binding proteins (RBPs), thereby affecting the function of the parental genes and alternative splicing (Liu et al., 2017; Zeng et al., 2017; Kristensen et al., 2019). Interestingly, increasing evidence shows that circRNA can encode proteins/peptides involved in cancer pathogenesis and progression (Legnini et al., 2017; Ye et al., 2018; Zheng et al., 2019). The unique characteristics and biological functions of circRNAs indicate that circRNA has the potential to be a promising biomarker for the diagnosis and prognosis of various diseases, as well as a therapeutic target (Kun-Peng et al., 2018; Zhou et al., 2019).

## The Characteristics of CircRNA

Accumulating evidence shows that circRNAs are ubiquitously distributed in eukaryotic cells, and have important characteristics and multiple biological functions, which make circRNAs the focus of interest in many scientific research fields, including ncRNA (Dong R. et al., 2017). (1) Since, unlike linear RNA, circRNA is a covalently closed loop with no 5′end cap or 3′poly (A) tail structure, it is not easily degraded by exonuclease and is more stable than linear RNA (Suzuki and Tsukahara, 2014). (2) CircRNAs are more diverse and abundant than their linear mRNA analogs. The expression of circRNA is often cell- type-, tissue- and developmental stage- specific (Patop et al., 2019; Huang et al., 2020). (3) Most circRNAs are evolutionarily conserved among different species, at both their sequence level and their pattern of expression (Jeck et al., 2012; Memczak et al., 2013; Salzman et al., 2013; Haddad and Lorenzen, 2019). (4) Except for intronic circRNAs that are sequestered in the nucleus, most ecircRNAs are exported to the cytoplasm in a size-dependent manner during their biogenesis (Huang et al., 2018). These characteristics of circRNAs indicate that they may play vital roles at both the transcriptional level and the post-transcriptional level, and may be useful in disease diagnosis.

## Biogenesis of CircRNAs

CircRNA is produced through a circularization process involving a canonical spliceosome-mediated precursor mRNA (pre-mRNA) back-splicing mechanism (Meng et al., 2017), which connects a downstream splice donor site (3′ splice site) to an upstream acceptor splice site (5′ splice site), and is modulated by RBPs and intronic complementary sequences. However, unlike in canonical (linear) splicing, in back-splicing the canonical cis-acting splicing regulatory elements and trans-acting splicing factors have different or even opposite activity. As briefly mentioned above, based on the synthesis of circRNAs from different locations of the genome splice junction from which circRNAs originate, circRNAs can be categorized into four types: ecircRNAs, intronic circRNAs, EIciRNAs, and intergenic circRNAs (Chen, 2016). Although, at present, the precise mechanism of circRNA biogenesis remains unclear, advances in sequencing technology, especially RNA-seq, have led the identification and characterization of numerous circRNAs, that resulted in significant progress regarding their biogenesis (Meng et al., 2017). It is known that ecircRNAs lack intronic sequences, are the most abundant type of circRNAs, predominantly localize to the cytoplasm and are formed by the reverse covalent binding of the splice donor site and the splice acceptor site of the pre-mRNA. Intronic circRNAs contain only intron sequences and include five subtypes, namely circular intronic RNAs (ciRNAs), excised group I introns, excised group II introns, excised tRNA introns, and intron lariats. EIciRNAs contain both intronic and exonic sequences as they are concurrently circularized by exons and introns, likely in similar manner to ecircRNAs. Intergenic circRNAs are another non-exonic circRNA type derived from the genomic interval between two genes and formed by two intronic circRNA fragments (ICFs) flanked by GT-AG splicing signals. As a result, three major models of the formation of ecircRNA or EIciRNA through a back-splicing mechanism have been proposed, namely lariat-driven circularization (exon skipping), intron pairing-driven circularization, and resplicing- or RBP-driven circularization (Ebbesen et al., 2016).

**FIGURE 1:**
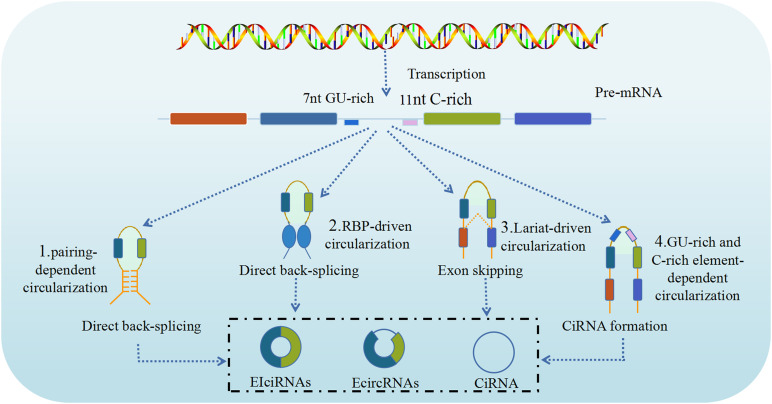
Biogenesis of circRNAs. CircRNAs are generated by back splicing of pre-mRNAs through different pathways (1, 2, 3, 4). CircRNAs can regulate the activities and functions of DNAs, RNAs and proteins in host cells, including: (1) Intron pairing-driven circularization; (2) RNA binding protein (RBP)-driven circularization; (3) Lariat-driven circularization; (4) GU-rich and C-rich element-dependent circularization (Cui et al., 2020).

The lariat-driven circularization model is associated with exon skipping, in which one or more exons of the transcript are skipped, generating a lariat consisting of both exons and introns. Then, the introns are removed to produce an ecircRNA. However, in some cases the introns are retained between the encircled and results in the formation of EIciRNA (Kristensen et al., 2019). In addition, if the activity of the debranching enzymes that control these lariat introns is reduced, these lariat introns escape subsequent degradation to form ciRNA through intron cyclization. The intron pairing-driven circularization (direct back-splicing) model (Zhang et al., 2014), the major pathway of ecircRNA production, is mostly associated with the pairing of flanking intronic complementary motifs (Alu elements) of the pre-mRNA, which induces circularization to form ecircRNA after the removal of introns. If there are inverted repeats, other than Alu elements, circRNA can also be generated via direct base-pairing of these inverted repeats. However, unlike ecircRNA, ciRNA formation is dependent on a conserved sequence near both sides of the spliceosome. CiRNA biogenesis relies on a 7-nt GU-rich element near the 5′ splice site and an 11-nt C-rich element near the branch point site (Petkovic and Müller, 2015). During direct back-splicing, the two elements bind into a lariat-like intermediate, containing the excised exons and introns, and are spliced by the spliceosome, generating lariats that undergo 3′ tail degradation, ultimately resulting in the production of ciRNAs (Petkovic and Müller, 2015). The RBP-driven circularization model is another biogenesis pathway to generate circRNAs by direct back-splicing driven by RBPs. Certain proteins, including quaking (QKI) and muscleblind like splicing regulator 1 (MBNL1, also termed MBL) proteins can bind to specific flanking intronic sequence motifs on linear pre-mRNA sequences and act as RBPs to link flanking introns, thus promoting circularization and subsequent circRNA generation (Conn et al., 2015). The process resembles the intron pairing-driven circularization model, except that in this case, after binding to specific putative binding sites, RBPs like MBNL1 dimerizes and brings the introns together, which leads to the circularization of the pre-mRNA and production (Figure 1).

## The Functions of CircRNAs

**FIGURE 2:**
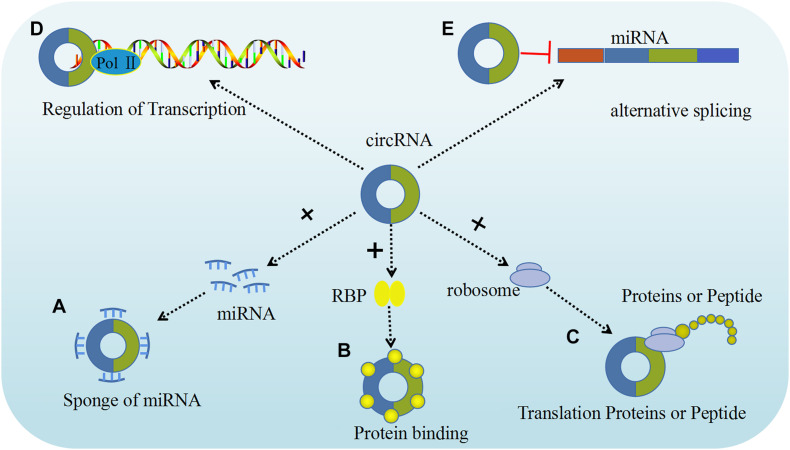
The functions of circRNAs. Five major functions of circRNAs have been identified, including: **(A)** Sponge of miRNA; **(B)** Proteins binding; **(C)** Translation proteins or peptide; **(D)** Regulation of transcription; **(E)** Alternative splicing (Yang et al., 2020).

It is well established that circRNAs can act as miRNA sponges by competing for miRNA binding sites, and diminish the effect of miRNA-mediated regulatory activities. The best characterized circRNA with miRNA sponge function, the human cerebellar degeneration related protein 1 antisense (CDR1as, also termed as ciRS-7) circRNA acts as a sponge for miR-671 and miR-7 to inhibit their expression *in vivo* (Piwecka et al., 2017). Experiments overexpressing CDR1as circRNA and using CDR1as gene knockdown demonstrated that overexpression of CDR1as circRNA increases the expression of miRNA targets, whereas its knockdown had the opposite effect, indicating that this circRNA be crucial for normal neuronal development. This type of *in vivo* models helps us gain a deeper understanding of the complex functions of circRNA in gynecological tumors. In human tumor cells, circRNA the following five possible functions in regulating different molecular pathways have been identified: miRNA sponging, protein binding, regulation of gene transcription or splicing, and translation of proteins or peptides (Figure 2).

## MicroRNA (miRNA) Sponging

Growing evidence has confirmed that circRNA regulates gene expression through competitive binding with miRNA, leading to its designation as a “miRNA sponge” (Chen et al., 2018). For example, the upregulation of circ_0103552 induced oncogenic activity in breast cancer cells, partly by directly sponging miR-1236, and is accompanied by a poor prognosis (Yang et al., 2019). Other findings indicate that circRNAs regulate the development of ovarian tumors through a variety of mechanisms, among which miRNA sponging is the most prominent. After being synthesized in the nucleus, ecircRNAs are transported to the cytoplasm where they mainly act as a miRNA sponge, while other circRNAs can interact with miRNA to transcribe or post-transcriptionally regulate gene expression (Chen, 2016). Recently, a study indicated that hsa_circ_0061140 is highly expressed in ovarian cancer (OC) cell. In addition, circ-FOXM1 becomes a competitive endogenous circRNA by binding to miR-370 (Chen et al., 2018; Yang et al., 2019). Several studies showed that circ-ITCH suppresses the Wnt/β-catenin pathway and thus its dysregulation is involved in the progression of various cancers (Hu C. et al., 2018). Circ-HIPK3 plays an important role in cancers by sponging multiple miRNAs, thereby promoting miRNA-124 and circFoxo3-mediated inhibition of the growth and survival of cancer cells (Teng et al., 2019) (Table 1).

**TABLE 1:** The expression and mechanisms of circRNAs in cervical cancer.

Circular RNAs	Cancers expression target gene	Clinical samples or cell lines	Mechanisms	References
has_circ_0018289 CC Up	_	35 pairs of cervical cancer tissue compared with adjacent normal tissue and cell lines	Hsa_circ_0018289 promote cervical cancer proliferation, migration and invasion via sponging miR-497.	Gao et al., 2017
circMTO1 CC Up	S100A1	HeLa and SiHa cells	Increase S100A1 expression by sponging miR-6983	Wright et al., 2009
hsa_circ_0000515 CC Up	ELK1	Hela, U14, SiHa, CaSki	IncreaseELK1 expression by sponging miR-326	

## CircRNAs Interact With and Bind to Proteins

Like linear RNAs, recent studies have shown that some circRNAs can also be used as RBP sponge to isolate the RBP by hiding the binding site of a specific protein, and competitively bind the protein and reduce protein activity. Some circRNAs, such as ciRS-7 and sex-determining region Y (SRY) circRNA (circ-SRY), can trigger the linearization and AGO2-mediated degradation of ciRS-7, which enables the release of the absorbed miRNA molecules and inhibit mRNA transcription (Capel et al., 1993). In addition, Du et al. (2016) found that circ-Foxo3 can competitively bind to p53 and mouse double minutes 2 homolog (MDM2), thereby decreasing the interaction between Foxo3 and MDM2 and enhancing Foxo3 activity, promoting MDM-induced p53 ubiquitination and degradation, ultimately leading to cell apoptosis (Huang et al., 2015; Li F. et al., 2015; Du et al., 2016; Wan et al., 2016). Circ-MBNL1 can bind to muscleblind like splicing regulator 1 (MBNL1, also termed MBL) protein; some studies have shown that due to the feedback loop mechanism, the biosynthesis of circ-MBNL1 will be affected by the level of MBNL1, and the high level of MBNL1 protein will also affect the process of converting MBNL1 pre-mRNA into circ-MBNL1 (Ashwal-Fluss et al., 2014). Furthermore, current evidence indicates that circRNAs can participate in the regulation of cancer pathogenesis by combining with various partners such as RBPs, including QBP, AGO, POL II, MBNL1, and other RBPs (Ashwal-Fluss et al., 2014; Du et al., 2016). For example, QKI interaction with circRNAs may lead to the regulation of epithelial-mesenchymal transition (EMT) during cancer progression. In addition, recent findings indicate that many circRNAs interact with HuR (a type of RBP) in cervical cancer (CC) cells. In addition, Circ-PABPN1 can bind to HuR, and HuR and circ-PABPN1 compete with PABPN1 mRNA (Dudekula et al., 2016). Despite extensive research on circRNAs, many remain unknown, and they may play key roles in cancer and could serve as biomarkers. According to these findings, it is safe to say that circRNAs have multiple functions in tumor development and progression.

## Regulation of Transcription and Alternative Splicing

Most circRNAs are located in the cytoplasm and regulate gene transcription. However, recent studies have reported that an EIciRNA accumulates at a transcription site and promotes the expression of the parental gene (Zhang et al., 2013). Knockout of a pair of EIciRNAs can inhibit the transcription of their host gene. Some EIciRNAs can interact with U1snRNPs (U1 small nuclear ribonucleoproteins), and the EIciRNA-U1 snRNP complex binds to RNA Pol II on the promoter of its parent gene, thereby enhancing gene expression. Once such RNA-RNA interaction is blocked, it destroys the interaction between EIciRNA and RNA Pol II and reduces the transcription of their host genes (Li P. et al., 2015). Moreover, certain circRNAs can compete with their linear counterparts against canonical pre-mRNA splicing, thus suppressing the expression of their host gene (Zhang et al., 2013; Wang et al., 2017). Cyclization and splicing compete with each other, leading to ecircRNAs to play a role in alternative splicing (Ji et al., 2019). Once back splicing occurs, it splices out internal exons, leading to alternative splicing. EIciRNA may be isolated from the translation start site, and thus truncated linear mRNA cannot be translated.

## Translating Proteins or Peptide

Although circRNAs are considered to be non-coding RNAs, accumulating data indicate that certain circRNAs are also translated into proteins. According to reports, circCTNNB1 produces a CTNNB1 isoform (i.e., β-catenin) containing 370 amino acids, which was found to be able to antagonize the GSK3β-induced β-catenin phosphorylation, thereby stabilizing the degradation of full-length β-catenin (Wang et al., 2015). In addition, recently numerous studies have shown that cyclic lncRNA-PINT may be translated into peptides to inhibit the proliferation of glioblastoma cells and the translation extension of oncogenes by capturing PAF1c (Abe et al., 2015). Unlike linear RNAs, some circRNAs prevent ribosomes from being recognized and translated into protein due to the lack of the 7-methylguanosine cap structure and poly(A) tail (Pamudurti et al., 2017). However, to date, almost no circRNA has been found to be translated into protein, so it is necessary to conduct further research in this field.

## The Expression, and Related Mechanisms of CircRNAs in Gynecological Cancers

### Expression of CircRNAs in Cervical Cancer

CC is the fourth most frequently diagnosed cancer and the fourth leading cause of cancer death in women worldwide in 2018 (Cohen et al., 2019). Human papilloma virus (HPV) infection is positively correlated with the occurrence and development of CC (Cohen et al., 2019). Recent study analyzed the expression of circRNAs in CC tissues. The analysis found that 45 different circRNAs were prominently expressed in CC tissues compared to control tissue samples, among which hsa_circ-0018289 was the most prominent. They found that knocking out this particular circRNA inhibited the proliferation and invasion of tumor cells. In addition, they also ascertained that hsa_circ_0018289 acts as a miRNA sponge and directly binds to miRNA-497 (Gao et al., 2017). Zheng et al. (2018) analyzed the circRNA expression profiles of CaSki CC cell line transfected with E7 sicirc-RNA and identified 526 abnormally expressed circRNAs. Subsequent bioinformatics analysis revealed that these HPV16/18-type E7 and E6 oncoproteins were necessary for the transformation and maintenance of the malignant phenotype of CC cells (Cai et al., 2018; Zheng et al., 2018). Chen et al. (2019) showed that the direct interaction between Circ-MTO1 and miR-6893 could rescue the invasion, migration and chemotherapeutic resistance of CC cells regulated by MTO1 Previous studies have shown that S100A1 promotes the progression of OC and hepatocellular carcinoma’ and circMTO1 was found to act as a sponge of miR-6893 to elevate the expression level of S100A1 in clinical CC (Wright et al., 2009). Moreover, Tang et al. (2019) reported that hsa_circ_0000515 was highly expressed in CC tissues and cells. Furthermore, hsa_circ_0000515 acts as a sponge of miR-326 and enhances ELK1 expression. ELK1, a transcription factor that belongs to the ETS family and ternary complex factor (TCF) subfamily, was found to play key roles in the regulation of cellular growth, differentiation, and survival (Dun and Gao, 2019). Inhibition of ELK1 inhibited cell cycle and promoted apoptosis. Thus, hsa_circ_0000515 plays a tumor promoting role in CC via hsa_circ_0000515/miR-326/ELK1 regulatory axis. Last but not least, they also suggested that hsa_circ_0000515 may be a promising target for CC treatment (Table 1).

### CircRNAs Suppress Tumor Progression

Currently, several circRNAs have been found to play different roles in a variety of cancer types. In cancer research, one of the best studied circRNAs is generated from the tumor suppressor gene FOXO3, a member of the forkhead family of transcription factors that has been found to induce apoptosis of cancer cells through a variety of biological processes. Circ-FOXO3 indirectly increases the expression of the linear mRNA of its host gene by binding to p53 and MDM2. The increase in circ-Foxo3 expression reduces the interaction between FOXO3 and MDM2, leading to the release of FOXO3, thereby increasing its activity, ultimately leading to increased apoptosis (Du et al., 2016; Kong et al., 2020). Hsa_circ_0001445 (also known as circ-SMARCA5) acts as a sponge for miR-620 and inhibits the progression of CC tumors, since its expression in CC cells is downregulated and its overexpression inhibits their proliferation, invasion and migration (Dai et al., 2018). Recently, circ-CLK3 was identified as a new type of circRNA overexpressed in CC. It has also been shown that inhibiting miR-320a and preventing its ability to inhibit the expression of the FoxM1 transcription factor promote cell proliferation, epidermal-mesenchymal transformation (EMT) and invasion of CC cells (211) (Hong et al., 2019) (Table 4).

### CircRNAs Promote Tumor Progression

Recently, a reported research study has shown that a new type of circRNA, namely circ-SLC26A4, is highly expressed in CC (Ji et al., 2020). Knockout of circ-SLC26A4 *in vitro* and *in vivo* was shown to inhibit tumor proliferation and invasion. Moreover, circ-SLC26A4 was found to target the 3′UTR of HOXA7 mRNA and sponge miR-1287-5p. Therefore, circ-SLC26A4 acts as a sponge of miR-1287-5p to promote the expression of HOXA7, which may potentially lead to new CC treatment strategies. In addition, circAMOTL1, which acts as a sponge for miR-485-5p, was found to enhance the expression of AMOTL1 (Ou et al., 2020). Moreover, Gao et al. (2017) found 45 upregulated circRNAs by analyzing 35 CC patients. Like other circRNAs, hsa_circ_0018289 exhibited carcinogenic effects on the progression of CC (He et al., 2020). Also, hsa_circ_0018289 was found to act as a sponge for miR-497, leading to increased cell proliferation of CC cells (He et al., 2020). Additionally, Mao et al. (2019) found that circ-EIF4G2 is overexpressed in CC cells and binds to miR-218, and at the same time, both can promote the expression of HOXA1 and enhance the progression of CC. Liu J. et al. (2018) showed that circ_8924 is overexpressed in CC cells and can bind to members of the miR-518d-5p/519-5p family. Therefore, they can promote the expression of the chromobox 8 (CBX8) axis (Liu J. et al., 2018) (Table 4).

## Circular RNAs and Ovarian Cancer

### Expression of CircRNAs in Ovarian Cancer

OC was one of the major causes of cancer death in women worldwide in 2020 (Siegel et al., 2020). Signaling pathways and dysfunction of cellular mechanisms significantly influence the progression and invasion of OC. The correlation and association between the regulation of circRNAs and OC have received much research interest. Ahmed et al. (2016) was the first to analyze circRNA expression in clinical ovarian tumors and found numerous differentially expressed circRNAs in tumor samples. They also detected differences in the miRNA and circRNA expression levels between primary and metastatic ovarian tumors. Regarding RNA exonuclease resistance and circRNAs stability, primary tumors were distinguished from metastatic lesions rather than mRNAs. Notably, in ovarian metastases, signaling pathways, such as STAT, AKT, NF-kB, TGF-B, ILK, HGF, and VEGF are commonly activated to produce linear RNA and angiogenesis signaling pathways that are negatively expressed in circRNAs. Hu J. et al. (2018) have demonstrated that circ-ITCH acts as a sponge for miR-145 to increase RASA1 expression and inhibit progression of OC cells. The miRNA sponge cycle in cells is a candidate for cancer diagnosis and treatment based on RNA. In contrast to the downregulated circ-ITCH expression, studies have indicated that the abnormal expression of circRNA in OC can play an oncogenic role (Hu J. et al., 2018). Recently, VPS13C-hsa_circ_001567 was shown to be able to promote the invasion and migration of OC cells, as well as enhance their invasion and migration ability. It was also shown that VPS13C-hsa_circ_001567 is involved in the VPS13C-has_circ_001567–miR-370– FOXM11 axis playing a role as the competing endogenous RNA (ceRNA) of the miR-370 sponge, thereby increasing the level of FOXM11 (Bao et al., 2019). This finding indicates that different circRNAs play opposite roles in the regulation of the biological behavior of OC cells by acting as tumor suppressor genes or oncogenes (Table 2).

**TABLE 2:** The expression and mechanisms of circRNAs in ovarian cancer.

Circular RNAs	Cancer expression target gene	Clinical samples or cell lines	Mechanisms	References
circITCH OC down	RASA1	20 paired OC tissues and adjacent normal tissues; CAOV3 and SKOV-3 cells	Increases FOXK2 expression by sponging miR-93-5p	Hu J. et al., 2018
VPS13C OC Down -has_circ_001567	FOXM11	20 paired OC tissues and adjacent normal tissues;SKOV-3 and OV-1063 cells	Increases N-cadherin expression And decreases E-cadherin expression	Bao et al., 2019
circ_0061140 OC Up	FOXM1	SKOV-3 and A2780 cells	Promotes EMT by increasing FOXM1 expression via sponging miR-370	Chen et al., 2018; Zou et al., 2018
circPLEKHM3 OC Down	BRCA1 DNAJB6KLF4	5 OC tissues and 5 normal ovarian epithelial tissues; A2780 and MDAH2274 cells	Inactivates the PI3K/AKT and Wnt/β-catenin pathways via promoting BRCA1, DNAJB6a and KLF4 expression by sponging miR-9	Zhang et al., 2019

### CircRNAs Suppress Tumor Progression

Previously, a number of studies showed that CDR1as can promote cancer in the various human cancer processes. Clinical studies have indicated that the expression level of CDR1as has a positive correlation with poor clinical prognosis, and it is a key prognostic indicator for lung cancer (Chen et al., 2019). However, some studies have shown that CDR1as is downregulated in OC tissues. In addition, CDR1as inhibits the function of OC cells by binding to miR-135b-5p. Other studies have shown that circ_100395 has a lower expression level in OC tissues than the corresponding non-cancerous tissues (Li et al., 2020). Furthermore, OC patients with a lower expression of circ_0078607 are more likely to have better prognosis. Therefore, circ_0078607 may be a crucial molecular target for patients with ovarian cancer. Also, circ_0078607 can function as a molecular sponge for miR-518a-5p, which promotes the inhibitory effect of miRNA on the expression of Fas, which is a membrane protein belonging to the tumor necrosis factor receptor superfamily (Zhang et al., 2020). Fas interacts with receptor Fas ligand (FasL), which induces a cascade of death signals and ultimately leads to cell apoptosis. According to the bioinformatics analysis results, Fas is the direct target of miR-518a-5p, thus we hypothesize that circ_0078607 acts as a sponge for miR-518a-5p and can increase the expression of Fas, thereby inhibiting the proliferation and invasion of OC cells and promoting their apoptosis. Accordingly, the circ_0078607/miR-518a5p/Fas axis can promote new aspects of the treatment of ovarian cancer patients (Zhang et al., 2020). Zhang et al. (2019) showed that Circ-PLEKHM3 downregulation can promote the progression of ovarian cancer cells; EMT induction promotes tumor metastasis; and CircPLEKHM3 upregulation can play the opposite role. Circ-PLEKHM3 can as a miR-9 sponge and since the ceRNA enhances the inhibitory effects of BRCA1, DNAJB6, and KLF4 on miR-9 target genes. The circ-PLEKHM3–miR-9 axis promotes the occurrence and metastasis of OC. Circ-PLEKHM3 expression is downregulated in OC. Patients with a low level of circ-PLEKHM3 have a poor prognosis (Zhang et al., 2019). Chen et al. (2018) found that hsa_circ_0061140 is overexpressed in OC cell lines And a Knockout of this gene can inhibit the progression of OC cells *in vivo* and *in vitro* by inhibiting the expression of fork head box M1 (FOXM1) by sponging miR-370 (Chen et al., 2018; Zou et al., 2018) (Table 4).

### CircRNAs Promote Tumor Progression

Zong et al. (2019) analyzed 79 cases of epithelial ovarian cancer tissue and 13 cases of normal ovarian tissue and measured the expression level of circ-WHSC1. Their results reveal that circ-WHSC1 is significantly overexpressed in OC and is significantly correlated with the degree of differentiation, which indicates that circ-WHSC1 may be significantly correlated with the progression of OC. Moreover, circ-WHSC1 was upregulated, while the expression of miR-1182 and miR-145 was upregulated in OC. The high expression of circ-WHSC1 may promote cell proliferation and inhibit cell apoptosis. Furthermore, circ-WHSC1 act as a sponge for miR-1182 and miR-145 to enhance the expression of MUC1 and hTERT. In other words, CircWHSC1 promotes the occurrence and development of OC through the miR-1182 and miR-145/MUC1 and hTERT axis, providing a promising target for the treatment of OC. Liu et al. (2020a) found that circ-GFRA1 is highly expressed in OC The downregulation of circ-GFRA1 inhibits cell proliferation and invasion and induce apoptosis. Additionally, circ-GFRA1 can play an important role by acting as a sponge for miR-449a. Therefore, circ-GFRA1 may be a potential diagnostic biomarker, and therapeutic target for OC (Liu et al., 2019). Finally, Xie et al. (2019) conducted a series of experiments to demonstrate that circ-EPSTI1 acts as a sponge for miR-942 to increase the expression of EPSTI1. In addition, another study showed that circEPSTI1 inhibited the occurrence and development of cancers, and induced apoptosis in OC, thus confirming its carcinogenic effect (Xie et al., 2019) (Table 4).

## Circular RNAs and Endometrial Cancer

### Expression of CircRNAs in Endometrial Cancer

Endometrial cancer (EC) is a common gynecological tumor in women all over the world. CircRNAs are highly conserved and stable non-coding RNAs, which have recently attracted considerable attention due to their potential function in cancer development (Li et al., 2018). They have multiple miRNA binding sites, which mediate their activity by competitively binding to target miRNAs, thereby inhibiting transcription of downstream genes (Patop and Kadener, 2018). However, the mechanism by which circRNAs regulate gene expression in EC remains unclear. Studies have shown that circ_0067835 is significantly increased in EC. Recently, circ_0067835 was found to be positively correlated with HMGA1 in EC (Liu et al., 2020a). HMGA1 is predicted to be a downstream target of miR-324-5p in EC and is identified as an important prognostic biomarker for EC. Circ_0067835 acts as a sponge for miR-324-5p to induce the expression of HMGA1.Accordingly, circ_0067835 can compete with miR-324-5p, leading to the overexpression of HMGA1, thereby inducing the progression of EC. In addition, the expression of hsa_circ_0002577 was found to be significantly upregulated in EC tissues, and its high expression was associated with advanced FIGO staging, lymph node metastasis and low overall survival rate of EC patients. Knockout of hsa_circ_0002577 significantly reduced the proliferation, and migration of EC cells *in vitro*, and reduced tumor growth *in vivo*. Therefore, hsa_circ_0002577 can play a vital role through the hsa_circ_0002577/miR-197/CTNND1/Wnt/β-catenin signaling pathway, which represents a new therapeutic option for developing EC therapeutics. Increasing evidence shows that by sequestering miRNAs, circRNAs play a key role in regulating gene expression (Shen et al., 2019). Circ_0109046 and HMGA2 were upregulated in EC tissues and cells, while miR-136 was downregulated. HMGA2 promotes tumor progression in gynecological cancers. Shi et al. (2020b) proposed that circ_0109046 acts as a sponge for miR-136 to enhance the expression of HMGA2, indicating that circ_0109046 may be a promising target for EC treatment. In addition, studies have shown that the expression level of Circ_PUM1 in EC tissues is significantly higher than that of normal tissues. The upregulation of circ_PUM1 promotes the proliferation, migration and invasion of EC cells. After knocking out circ_PUM1, the tumorigenic ability of EC cells is reduced. Circ_PUM1 can compete with miR-136, leading to the up-regulation of NOTCH3, thereby promoting EC development (Zong et al., 2020) (Table 3).

**TABLE 3:** The expression and mechanisms of circRNAs in ovarian cancer.

Circular RNAs	Cancer expression target gene	Clinical samples or cell lines	Mechanisms	References
circ_0067835 EC Up	HMGA1	10 Endometrial cancer tissues and 10 normal endometrial tissues;HEC1-B and RL95-2 cells	Increases HMGA1 expression by sponging miR-324-5p	Liu et al., 2020a
hsa_circ_0002577 EC Up	CTNND1	36 paired EC tissues and adjacent normal endometrial tissues; ECC-1 and HEC-1-A cells	Increases Wnt/β-catenin pathways via promoting CTNND1 expression by sponging miR-197	Shen et al., 2019
circ_0109046 EC Up	HMGA2	44 Endometrial cancer tissues and 44 normal endometrial tissues;HEC1-A, KLE and Ishikawa cells	Increases HMGA2 expression by sponging miR-136	Shi et al., 2020a
circPUM1 EC Up	NOTCH3	69 paired EC tissues and adjacent normal endometrial tissues; HEC-1B CELLS and Ishikawa human endometrial carcinoma cells	Increases NF-κB/MMP2 expression by sponging miR-615-5p/miR-6753-5p	Zong et al., 2020

**TABLE 4:** CircRNAs suppress and promote gynecological tumors progression.

Circular RNAs	Gynecological cancers	miRNA	Cancer expression target gene	Function	References
hsa_circ_0001445 (circ-SMARCA5)	CC	miR-620	−	−	Dai et al., 2018
circ-CLK3	CC	miR-320a	FoxM1	−	Dai et al., 2018
circ-SLC26A4	CC	miR-1287-5p	HOXA7	+	Ji et al., 2020
circAMOTL1	CC	miR-485-5p	AMOTL1	+	Ou et al., 2020
circ-EIF4G2	CC	miR-218	HOXA1	+	Mao et al., 2019
circ_8924	CC	miR-518d-5p/519-5p	CBX8	+	Liu J. et al., 2018
circCDR1as	OC	miR-135b-5p	−	−	Chen et al., 2019
circ_0078607	OC	miR-518a-5p	Fas	−	Zhang et al., 2020
circ-PLEKHM3	OC	miR-9	BRCA1, DNAJB6,KLF4	−	Zhang et al., 2019
hsa_circ_0061140	OC	miR-370	FOXM1	−	Chen et al., 2018; Zou et al., 2018
circ-WHSC1	OC	miR-1182 miR-145	MUC1/hTERT	+	Zong et al., 2019
circ-GFRA1	OC	miR-449a	−	+	Liu et al., 2020a
circEPSTI1	OC	miR-942	EPSTI1	+	Xie et al., 2019
circWHSC1	EC	miR-136	NPM1	+	Liu et al., 2020a
circTNFRSF21	EC	miR-1227	MAPK13/ATF2	+	Liu et al., 2020a
hsa_circ_0061140	EC	miR-149-5p	STAT3	+	Liu et al., 2020a

**Promote tumor progression (+); suppress tumor progression* (−*).**

### CircRNAs Promote Tumor Progression

Some previous studies have shown that circWHSC1 is highly expressed in (EC) and promotes EC. Liu et al. (2020d) collected a total of 26 normal endometrial tissues and 32 EC tissues, which were confirmed pathologically. They found that overexpression of circWHSC1 promoted cell proliferation, migration and invasion, and inhibited apoptosis (Liu et al., 2020d). In addition, it has been reported that circWHSC1 acts as a sponge for miR-136 and targets NPM1 mRNA to negatively regulate the progression of EC. NPM1 is highly expressed in EC and has a positive correlation with the clinical stage and histological grade of EC. A recent study in 28 tumor samples and adjacent normal tissues of EC patients showed that circTNFRSF21 rescues the MAPK13-ATF2 signaling pathway activity by acting as a miR-1227 sponge in EC cells. The high expression level of circTNFRSF21 promoted EC cell growth, cell cycle progression and *in vivo* tumor growth. Thus, circTNFRSF21-miR-1227-MAPK13/ATF2 axis may be a promising target in EC treatment (Liu et al., 2020b). Liu et al. (2020b) showed that hsa_circ_0061140 acts as a sponge for miR-149-5p and as an oncogenic circRNA in EC. In addition, it has also been confirmed that STAT3 is a downstream target of miR-149-5p. Therefore, hsa-circ_0061140 promotes the progression and migration of EC by regulating miR-149-5p and STAT3 (Liu et al., 2020c) (Table 4).

## The Biomarkers and Therapeutic Targets of CircRNAs in Gynecological Cancers

### CircRNAs as Promising Biomarkers of Cervical Cancer Prognosis

**FIGURE 3:**
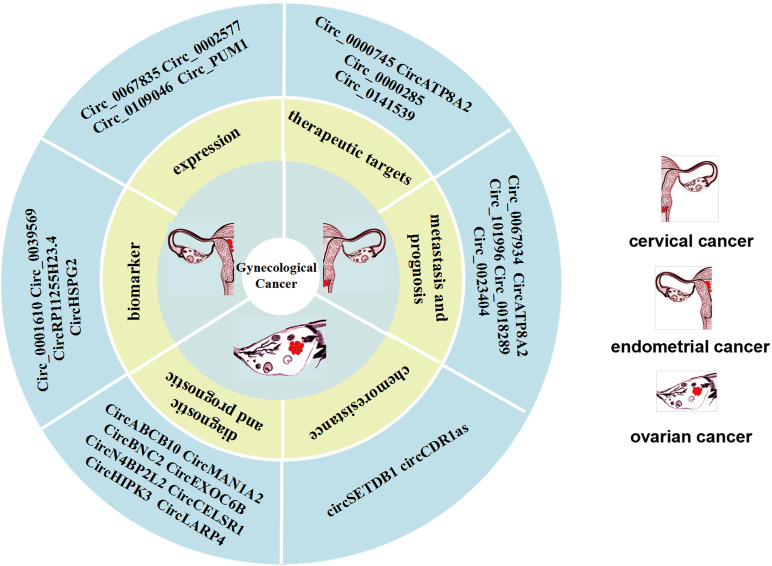
CircRNAs can be used as biomarkers of cervical cancer (CC) metastasis and prognosis as well as potential therapeutic targets. CircRNAs can be used as biomarkers of ovarian cancer (OC) diagnosis and prognosis, as well as chemoresistance. Expression of CircRNAs are expressed in endometrial cancer (EC) and are potential biomarkers for endometrial cancer.

Compared with paired normal tissues, circ_0067934 is highly expressed in CC tissues and cell lines. The upregulation of circ_0067934 is also related to positive lymphatic metastasis of CC patients, which indicates that it can be used as a promising biomarker for CC metastasis. Compared with adjacent normal tissues, circ_0018289 is highly expressed in CC tissues, and is strongly associated with decreased overall survival rate (He et al., 2020). This suggests that circ_0018289 may be a promising biomarker for prognostic evaluation. Moreover, it has been shown that hsa_circ_0023404 is overexpressed in CC tissues and cell lines (Zhang et al., 2018). This finding indicates that the overall survival rate of patients with high expression level of hsa_circ_0023404 is lower than that of patients with low expression level of hsa_circ_0023404. The possible related mechanism is that hsa_circ_0023404 sponges miR-136. Also, hsa_circ_0023404 induces the activation of the related protein Yes-associated protein (YAP) pathway, which leads to the progression of CC (Zhang et al., 2018). Recently, Song et al. (2019) found that hsa_circ_101996 is upregulated in CC and is related to tumor stage, size, lymph node metastasis and poor prognosis. Moreover, circ_0067934 sponges miR-545 and is correlated with advanced cancer, lymph node metastasis and metastasis, as well as poor prognosis (Hu C. et al., 2018; Song et al., 2019). In addition, circ-ATP8A2 is also upregulated in CC cells, and the circATP8A2/miR-433/EGFR axis plays a critical role in the progression of CC (Mao et al., 2019) (Figure 3).

### CircRNAs as Potential Therapeutic Targets in Cervical Cancer

A very recent study has shown that has_circ_0000745 is overexpressed in CC patients, and its high expression is correlated with poor tumor differentiation and vascular invasion. Knocking out hsa_circ_0000745, upregulated the expression of cadherin 1, which can inhibit the progression of CC cells, indicating that this circRNA could be an ideal target candidate for the development of CC therapy (Jiao et al., 2019). Other studies have shown that post-transcriptionally circ-ATP8A2 acts as a sponge for miR-433 to inhibit the expression of epidermal growth factor receptor (EGFR). Studies in a nude mouse model revealed that the knockout of circ_0000285 can clearly inhibit the occurrence and migration of CC. In other words, circ_0000285 can promote the progression and metastasis of CC (Chen et al., 2019). Other studies found that the expression of circRNA_8924 in CC tissue samples was significantly higher than that in adjacent non-tumor tissue samples, and was correlated with FIGO staging, myometrial invasion, and tumor size (Liu J. et al., 2018). Also, circRNA_8924 was found to act as a sponge for miR518d-5p/519-5p to elevate CBX8 expression and inhibit progression of CC cells and promote apoptosis of CC cells (Figure 3).

### CircRNAs as Biomarkers of Ovarian Cancer Diagnostic and Prognostic

#### CircRNAs as Novel Serum Biomarkers of Ovarian Cancer Diagnostic and Prognostic

CircRNAs can reflect the progression of cancer in clinical practice and can be used to predict the prognosis of OC patients, thus it has the potential to act as a biomarker. Research on circulating cell-free circRNA extracted from blood is a hot topic in the field of diagnostic biomarkers (Chen et al., 2019; Fan et al., 2019). For example, Fan et al. (2019) collected 414 serum samples, including samples from 121 healthy controls, and found that circMAN1A2 was significantly upregulated in the serum samples of patients with NPC, oral cancer, thyroid cancer, ovarian cancer, and lung cancer and had good clinical diagnostic value. Thus, they suggested that circMAN1A2 could be a serum biomarker for malignant tumors, providing important insights into diagnostic approaches for malignant tumors (Fan et al., 2019). In addition, the circulating circ-ABCB10 can also serves as diagnostic biomarkers of OC, based on the finding that the high expression level of circ-ABCB10 is correlated with the lower overall survival rate of OC patients (Chen et al., 2019) (Figure 3).

#### CircRNAs as Biomarkers of Ovarian Cancer Tissue Diagnostic and Prognostic

In addition, Ning et al. (2018) found that the circ-EXOC6B, circ-N4BP2L2 and circ-CELSR1, are clearly dysregulated in OC tissues, and thus may represent promising OC biomarkers. It is of great importance to evaluate the prognosis of doctors of the OC patients. It has been reported that circ-BNC2 can distinguish early stage ovarian cancer patients from benign and healthy subjects, and may be a specific diagnostic biomarker for OC (Hu et al., 2019). The low expression of circ-EXOC6B and circ-N4BP2L2 in OC patients is closely related to the overall survival rate and disease-free survival rate. However, it has been demonstrated that several circRNAs can suppress tumors in OC patients and their prognosis evaluated. The Circ-HIPK3 was found to be overexpressed in OC tissues and its high expression was significantly correlated with lymph node infiltration, FIGO stage and poor prognosis in OC patients (Liu N. et al., 2018). Recent studies have shown that the expression of circ-LARP4 is significantly downregulated in OC tissues, and is correlated with FIGO stage, progression and lymph node metastasis. Therefore, circ-LARP4 may be a promising marker for predicting the prognosis of OC patients (Zou et al., 2018) (Figure 3).

### CircRNAs in Ovarian Cancer Chemoresistance

Chemoresistance and recurrence of OC have become a burden for effective management of the disease. It has been reported that there are many subtypes of cancer associated with chemoresistance and a circRNA. Patients with OC often develop chemotherapy resistance, which makes it unlikely to achieve satisfactory treatment outcomes with chemotherapy. Wang et al. (2019) found that the higher circ-SETDB1 expression level in serum is significantly correlated with lymph node metastasis and clinical stages, and can be used as an important indicator to distinguish patients from healthy subjects. It should be noted that in that study the circ-SETDB1 serum expression level of patients with primary chemotherapy resistance was significantly increased, indicating that serum circ-SETDB1 is likely an important indicator of the OC chemotherapy response and recurrence. Zhao et al. (2019) compared cisplatin-resistant and sensitive OC tissues at the expression level of circRNAs and found that expression level of CDR1as in tissues and cells from patients with cisplatin resistance was generally low (Zhao et al., 2019). In other studies, an inhibitory effect of CDR1as on OC cells. These studies showed that CDR1as can enhance the sensitivity of OC to platinum through the miR-1270/SCAI signaling pathway (Figure 3).

### CircRNAs as Potential Biomarker in Endometrial Cancer

In recent years, circRNAs have been used as a promising biomarker and the mechanism of action of certain circRNAs in EC cells and tissues have been studied. Numerous studies have shown that there are 75,928 dysregulated circRNAs in EC cells. There are 62,167 circRNAs expressed in EC cells, which are significantly upregulated or downregulated compared to their expression in normal endometrial tissue (Ye et al., 2019). Both Hsa_circ_0039569 and hsa_circ_0001610 are relatively unique circRNAs., Although their expression levels have nothing to do with myometrial invasion, grade 1–2 EC, lymph node metastasis, tumor size, FIGO stage, and patient age, they are closely correlated with tumor differentiation. Some studies indicate that the hsa_circ_0039569 interacts with the hsa-miR-542-3p/hsa-let-7c-5p axis, which has a low expression level in grade 3 EC. In other words, hsa_circ_0039569 inhibits the expression of hsa-miR-542-3p/hsa-let-7c-5p, which may be a promising biomarker in EC (Xu et al., 2018). According to reported research, RP11255H23.4 and HSPG2 are expressed in normal endometrial tissues, but not in EC tissues (Shi et al., 2020a). Moreover, the corresponding miRNA expression level is also elevated state in normal tissues, which indicates that circRNAs can competitively combine with related miRNAs to promote the progression of EC. Together the above research results showed that circRNAs may be promising biomarkers and therapeutic targets for the diagnosis of EC (Figure 3).

## Conclusion

In conclusion, as described in this review, dysregulation of the expression of circRNA genes is considered to be one of the main mechanisms driving tumorigenesis and progression. A large amount of research data indicates that circRNAs have important roles in the progression of various cancers. The latest research shows that abnormal circRNA expression can have an important impact on the occurrence and development of gynecological cancers through a miRNA sponge mechanism. EIciRNAs are very stable, so they have great potential for application in the diagnosis or therapeutic intervention of tumors. Furthermore, certain circRNAs may be ideal for the treatment of tumors. With the advances and application of high-throughput sequencing technology, researchers have identified increasing an increasing number of circRNAs. However, their role and mechanism in gynecological tumors are still unclear. Moreover, the recent research sample population usually comes from a single research center and the number of samples is relatively small. Furthermore, due to the complexity of the tumor, not many exact functions have be found. Therefore, the use of more readily available clinical sample types (such as serum, urine) will improve the reliability of the research results. At present, most research focuses on the sponge function of circRNAs, and to a certain extent, the research on other functions is ignored. The pathogenesis of tumors is complex and variable, thus, the mechanisms of action of circRNAs in tumors have not been thoroughly studied, and further research on this topic is needed. Based on the conclusions of current clinical and experimental studies, this article summarized the potential of circRNAs as new biomarkers of the diagnosis and treatment of gynecological cancers. However, research on circRNAs in gynecological tumors is still in the early stages, further research is needed to broaden the application potential of circRNAs.

## Author Contributions

YM, LZ, YG, WZ, QZ, and YX performed literature searches and selected the studies and reviews discussed in the manuscript. The first draft of the manuscript was prepared by YM, LZ, YG, WZ, and QZ made subsequent amendments. YX revised the manuscript. All authors read and approved the final manuscript and contributed to the conception of this review.

## Conflict of Interest

The authors declare that the research was conducted in the absence of any commercial or financial relationships that could be construed as a potential conflict of interest.
